# *Azotobacter vinelandii s*caffold protein NifU transfers iron to NifQ as part of the iron-molybdenum cofactor biosynthesis pathway for nitrogenase

**DOI:** 10.1016/j.jbc.2024.107900

**Published:** 2024-10-22

**Authors:** Emma Barahona, Juan Andrés Collantes-García, Elena Rosa-Núñez, Jin Xiong, Xi Jiang, Emilio Jiménez-Vicente, Carlos Echávarri-Erasun, Yisong Guo, Luis M. Rubio, Manuel González-Guerrero

**Affiliations:** 1 Centro de Biotecnología y Genómica de Plantas, Universidad Politécnica de Madrid, Instituto Nacional de Investigación y Tecnología Agraria y Alimentaria, Madrid, Spain; 2 Departamento de Biotecnología-Biología Vegetal, Escuela Técnica Superior de Ingeniería Agronómica, Alimentaria y de Biosistemas, Universidad Politécnica de Madrid, Madrid, Spain; 3 Department of Chemistry, Carnegie Mellon University, Pittsburgh, Pennsylvania, USA; 4 Department of Biochemistry, Virginia Polytechnic Institute, Blacksburg, Virginian, USA

**Keywords:** iron, iron-sulfur protein, molybdenum, nitrogen fixation, nitrogenase

## Abstract

The *Azotobacter vinelandii* molybdenum nitrogenase obtains molybdenum from NifQ, a monomeric iron-sulfur molybdoprotein. This protein requires an existing [Fe-S] cluster to form a [Mo-Fe_3_-S_4_] group, which acts as a specific molybdenum donor during nitrogenase FeMo-co biosynthesis. Here, we show biochemical evidence supporting the role of NifU as the [Fe-S] cluster donor. Protein-protein interaction studies involving apo-NifQ and as-isolated NifU demonstrated their interaction, which was only effective when NifQ lacked its [Fe-S] cluster. Incubation of apo-NifQ with [Fe_4_-S_4_]-loaded NifU increased the iron content of the former, contingent on both proteins being able to interact with one another. As a result of this interaction, a [Fe_4_-S_4_] cluster was transferred from NifU to NifQ. In *A. vinelandii*, NifQ was preferentially metalated by NifU rather than by the [Fe-S] cluster scaffold protein IscU. These results indicate the necessity of co-expressing NifU and NifQ to efficiently provide molybdenum for FeMo-co biosynthesis when engineering nitrogenase in plants.

Nitrogenases catalyze the reduction of N_2_ to NH_3_ in an energetically expensive process (1). These enzymes, only present in some bacteria and archaea, are two-component oligomeric metalloprotein complexes made up of a dinitrogenase (component I) and a dinitrogenase reductase (component II) (2). Component I of the molybdenum nitrogenase, the most widespread, is a heterotetramer formed by two NifD, two NifK proteins, and two pairs of different metalloclusters. The iron-molybdenum cofactor (FeMo-co; [Fe_7_-S_9_-C-Mo-*R*-homocitrate]) is present at the active site of each NifD subunit, while the [Fe_8_-S_7_] P-cluster is at the interface of each NifD and NifK subunits (3, 4). Component II is a homodimer encoded by *nifH*. This protein contains a single [Fe_4_-S_4_] cluster bridging the two identical subunits and two sites for Mg^2+^-ATP binding and hydrolysis (1). Electrons provided to NifH are transferred from its [Fe_4_-S_4_] cluster through the P-cluster of NifDK to FeMo-co, where N_2_ is reduced (5, 6). Therefore, for nitrogenase to function, these metal cofactors must be assembled, protected from O_2_, and transferred to the apo-enzymes, a tightly regulated process that requires additional proteins (5). Among them, NifU and NifQ are the known points from where iron and molybdenum, respectively, are specifically directed toward nitrogenase cofactor assembly (5).

NifU is a 33 kDa homodimer with a permanent [Fe_2_-S_2_] cluster per subunit (7). It serves as a scaffold to synthesize [Fe_4_-S_4_] groups, receiving iron from unknown donor(s) and sulfide from NifS, a 43 kDa cysteine desulfurase (8, 9). These groups are transiently assembled in the N- and C-terminal domains of NifU and are subsequently transferred to target proteins. For example, the transfer of [Fe_4_-S_4_] clusters from NifU to apo-NifH to convert the latter to active holo-NifH is well studied (10). NifU is also involved in FeMo-co biosynthesis, providing the [Fe_4_-S_4_] clusters required as a substrate for NifB-co assembly by NifB (11). These data indicate a pivotal role of NifU in Fe-S cluster assembly and transfer to the different enzymes involved in nitrogenase cofactor assembly.

In many nitrogen-fixing prokaryotes, NifQ provides molybdenum for FeMo-co assembly. This protein is present in all diazotrophic species of Proteobacteria, except for some *Rhizobia* (12). *Azotobacter vinelandii* and *Klebsiella pneumoniae nifQ* mutant strains are impaired in nitrogen fixation unless molybdate levels are significantly increased in the growth medium (13, 14). NifQ is a 22 kDa monomeric [Fe-S] cluster-containing molybdoprotein (15). It has been shown that NifQ synthesizes a [Mo-Fe_3_-S_4_]^3+^ group using a [Fe_3_-S_4_]^+^ precursor, although the mechanism of synthesis is still unknown (16). Subsequently, NifQ will transfer molybdenum or [Mo-Fe-S] clusters to a NifEN/NifH complex for molybdenum integration into FeMo-co (15).

The origin of the [Fe-S] cluster precursor for NifQ is currently unknown. Given the central role of NifU as the scaffold in which [Fe-S] clusters destined for some nitrogenase components are assembled (10, 11), it can be hypothesized that it is also the primary source for the NifQ clusters. Supporting this role, here we report that NifU transfers a [Fe_4_-S_4_] cluster to NifQ through direct protein-protein interaction.

## Results

### NifU and NifS co-elute with NifQ

**Figure 1:**
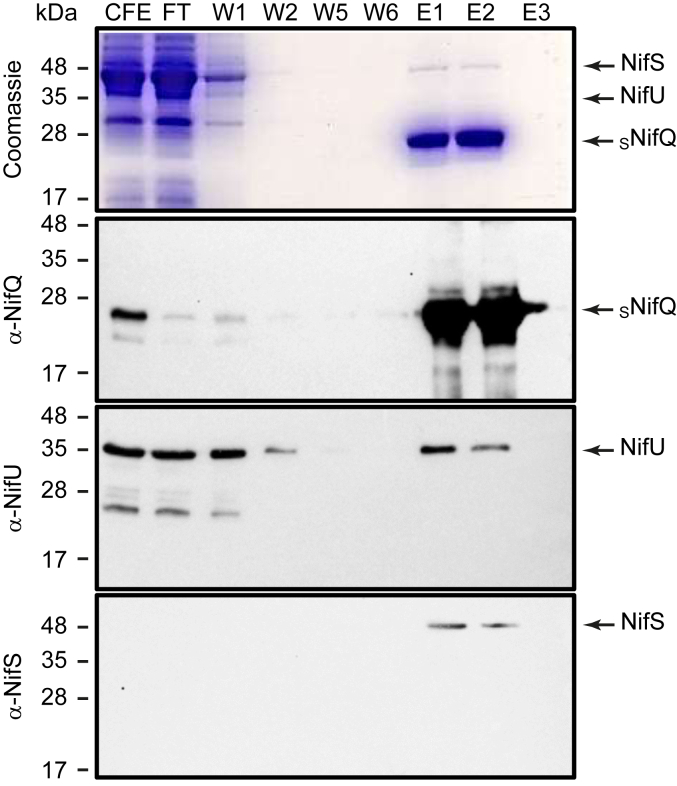
**NifU and NifS copurify with**_**S**_**NifQ from *E. coli* extracts expressing *nifQ, nifU,* and *nifS*.***Top panel* shows the Coomassie staining of an SDS-PAGE of cell free extract (CFE), flowthrough (FT), wash (W1-W6) and elution (E1-E3) fractions of the extracts passed through a Streptactin column. The remaining panels show immunoblots of the same fractions developed with anti-NifQ, anti-NifU, or anti-NifS antibodies. Images show a representative assay (n = 2). Uncropped immunoblots and gels are shown in Fig. S5.

To determine whether NifQ can interact with the [Fe-S] cluster biosynthesis branch of the FeMo-co biosynthetic pathway, an N-terminal Strep-tagged *A. vinelandii* NifQ (_S_NifQ) was expressed in an *Escherichia coli* strain that already produced the *A. vinelandii* NifU and NifS proteins. After Nif protein induction and cell lysis, _S_NifQ was purified by StrepTactin Affinity Chromatography (STAC) performed under anaerobic conditions. As expected, _S_NifQ was the most abundant protein in the eluted fractions, as evidenced by Coomassie blue staining of SDS-gels, as well as the immunodetection of NifQ with specific antibodies (Fig. 1). To determine whether NifU and NifS were among these additional bands, specific antibodies raised against either protein were used for immunoblotting. As shown in Fig. 1, both NifU and NifS co-eluted with sNifQ. These were not the result of unspecific interaction of NifU and/or NifS with the purification resin since both proteins were not detected in the elution fractions when sNifQ was not expressed in this *E. coli* strain (Fig. S1).

### The interaction between NifQ and NifU is NifS independent and apo-NifQ dependent

**Figure 2:**
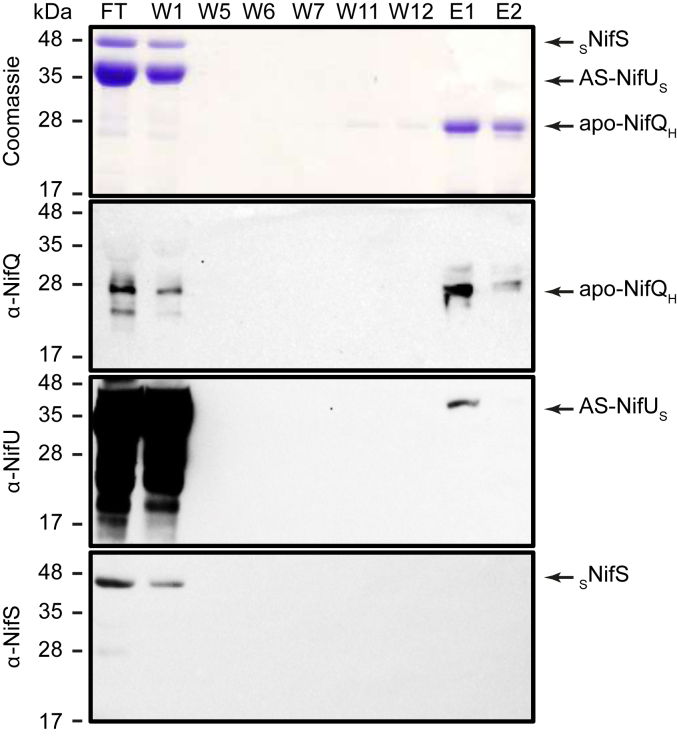
**AS-NifU_S_ interacts with apo-NifQ_H_.***Top panel* shows the Coomassie staining of an SDS-PAGE of flowthrough (FT), wash (W1-W12) and elution (E1-E2) fractions of a mixture solution containing AS-NifU_S_, _S_NifS, and apo-NifQ_H_ passed through a Ni^2+^ column. The remaining panels are the immunoblots of the same fractions developed with anti-NifQ, anti-NifU, or anti-NifS antibodies. Images show a representative assay (n = 3). Uncropped immunoblots and gels are shown in Fig. S6.

The co-purification of NifU and NifS with NifQ from crude extracts of recombinant *E. coli* cells could be the consequence of direct interactions among these three proteins, or in combination with endogenous *E. coli* proteins. To discriminate between these two possibilities, NifQ was purified using a (His)_6_ tag as an apo-form (apo-NifQ_H_) containing less than one iron atom per monomer (Table 1). On the other hand, the “*as purified*” (AS) NifU_S_ contained 2.4 iron atoms per monomer (Table 1). Apo-NifQ_H_, and the Strep-tagged AS-NifU_S_ and _S_NifS, were incubated together for 5 min under anaerobic conditions and subsequently loaded onto a Ni-NTA column. As shown in Fig. 2, apo-NifQ_H_ was properly captured by the resin, and the protein was eluted by applying 150 mM imidazole. Most soluble AS-NifU_S_ was detected in the flowthrough and early wash fractions, but a significant amount, around 10% of the total protein, co-eluted with apo-NifQ_H_. AS-NifU_S_ presence in the elution fractions was due to apo-NifQ_H_; when NifQ was not present, no NifU was detected in the eluates (Fig. S2). These results confirm the apo-NifQ/AS-NifU interaction without additional proteins being required. On the contrary, _S_NifS was only detected in the flowthrough and initial wash fractions (Fig. 2), suggesting that NifS was not necessary for the apo-NifQ/AS-NifU interaction.

**Table 1:** Proteins used in this work

Protein	Name	Tag/Position	Organism	Fe/monomer	Mo/monomer	Source
NifQ	apo-_S_NifQ	Strep/N-t	*E. coli* BL21	0.56 ± 0.01	0.01 ± 0.001	This work
	apo-NifQ_H_	6xHis/C-t	*E. coli* BL21	0.04 ± 0.01	0.01 ± 0.011	This work
	holo-_S_NifQ	Strep/N-t	*E. coli* BL21	2.80 ± 0.32	0.01 ± 0.004	This work
	AS-NifQ_H_	6xHis/C-t	*E. coli* BL21	1.39 ± 0.40	0.00 ± 0.000	This work
	AS-NifQ_H_	6xHis/C-t	*A. vinelandii* UW3000	2.58 ± 0.07	0.39 ± 0.17	This work
	AS-NifQ_H_	6xHis/C-t	*A. vinelandii* MGGAV1 (UW3000 Δ*nifU*)	0.8 ± 0.05	0.09 ± 0.03	This work
NifU	AS-_H_NifU	6xHis/N-t	*E. coli* BL21	2.72 ± 0.46	-	(17)
	AS-NifU_S_	Strep/C-t	*E. coli* BL21	2.38 ± 0.28	-	This work
	R-NifU_S_	Strep/C-t	*E. coli* BL21	6.08 ± 0.86	-	This work
	R-_H_NifU	Strep/N-t	*E. coli* BL21	4.25 ± 0.77	-	This work
NifS	_S_NifS	Strep/N-t	*E. coli* BL21	-	-	This work
IscU	R-IscU_S_	Strep/C-t	*E. coli* BL21	4.45 ± 0.74	-	This work

Data are the average iron content per protein monomer ± SD calculated for apo-_S_NifQ (n = 3), apo-NifQ_H_ (n = 8), holo-_S_NifQ (n = 2), holo-NifQ_H_ (n = 2), AS-NifQ_H_ (n = 2), AS-_H_NifU (n = 2), AS-NifU_S_ (n = 2), R-NifU_S_ (n = 2), R-_H_NifU (n = 2), and R-IscU_S_ (n = 3). S indicates Strep-tagged protein; H indicates 6xHis-tagged protein; AS indicates as isolated protein; and R indicates proteins with *in vitro* reconstituted [Fe-S] clusters.

**Figure 3:**
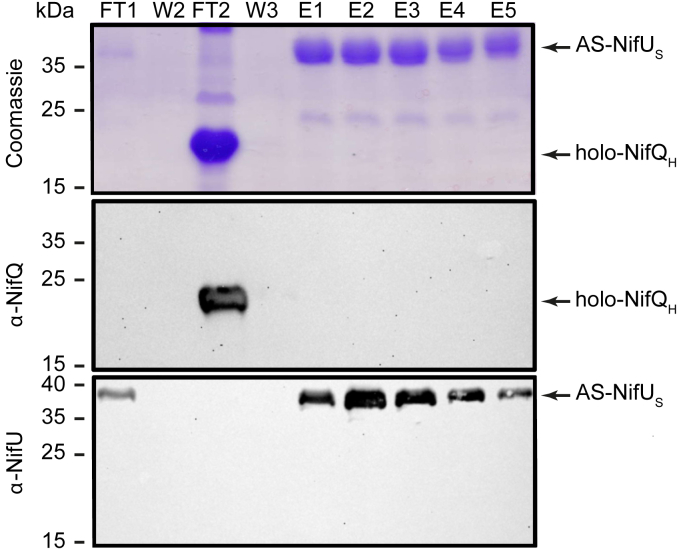
**Holo-NifQ**_**H**_**does not co-elute with AS-NifU**_**S**_. *Top panel* shows the Coomassie staining of an SDS-PAGE of holo-NifQ_H_ pull-down assay using AS-NifU_S_ as bait. FT1 represents the flow-through fraction obtained after loading AS-NifU_S_ onto the Strep-column. W2 represents the second wash fraction. FT2 represents the flow-through fraction obtained after loading holo-NifQ_H_ onto the column. W3 represents the third wash fraction after passing holo-NifQ_H_ over an AS-NifU_S_-charged column. E1, 2, 3, four and five represent elution fractions. Images show a representative assay (*n* = 4). Uncropped immunoblots and gels are shown in Fig. S7.

Considering that the metalation state of NifQ might influence the interaction with NifU, co-purification assays were carried out between NifQ_H_ in its holo-state and AS-NifU_S_. Holo-_S_NifQ contained 2.8 iron atoms per monomer (Table 1). In contrast to what was observed using apo-NifQ_H_, no interaction with NifU was observed (Fig. 3). These data suggest that when NifQ is already occupied by a [Fe-S] cluster, the interaction with NifU is non-existent or reduced below our detection limit.

### The iron content of NifQ increases after the interaction with NifU

**Figure 4:**
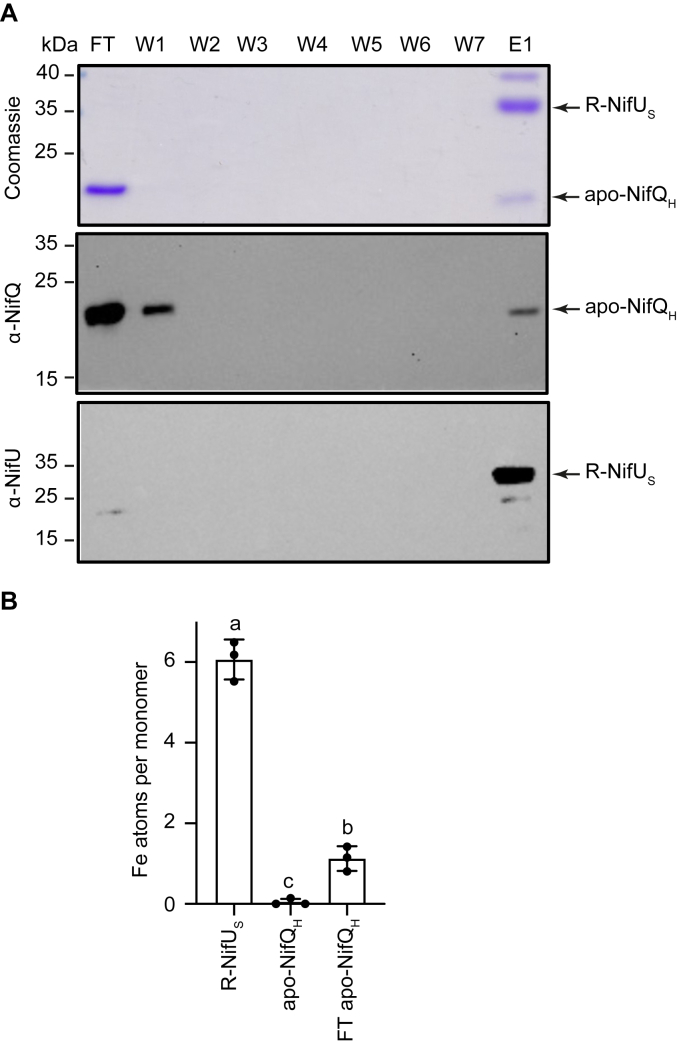
**NifU_S_ transfers iron to apo-NifQ_H_.***A*, *top panel* shows the Coomassie staining of an SDS-PAGE of flowthrough (FT), wash (W1-W7) and elution (E1) fractions of a mixture solution containing R-NifU_S_ and apo-NifQ_H_ passed through a Strep-tactin column. The remaining panels are the immunoblots of the same fractions developed with anti-NifQ or an anti-NifU antibody. Uncropped immunoblots and gels are shown in Fig. S8. *B*, iron content per monomer of isolated proteins prior to interaction, and in the FT fraction obtained from passing through a Strep-tactin-column a solution in which apo-NifQ_H_ was incubated for 5 min with R-NifU_S_. Bars represent the average ± SD (n = 3). Different letters indicate statistically significant differences (*p* < 0.01). The dots represent each individual data point.

The fact that the interaction between NifQ and NifU is contingent upon the iron content of NifQ is suggestive of a process in which iron would be transferred from NifU to NifQ. This possibility was tested by determining the iron transfer from one protein to the other. Apo-NifQ_H_ was incubated with a reconstituted (R) NifU_S_ that contained a higher complement of transient [Fe_4_S_4_] clusters, as indicated by a 6.1 iron:monomer ratio (Table 1). These proteins were incubated for 5 min to allow for iron transfer and then separated using a Strep-column. The interaction between apo-NifQ_H_ and R-NifU_S_ was still observed (Fig. 4*A*), but in the flowthrough only apo-NifQ_H_ could be detected. As shown in Fig. 4*B*, incubation with R-NifU_S_ significantly increased the iron content in NifQ_H_ to around 1:1 M ratio.

**Figure 5:**
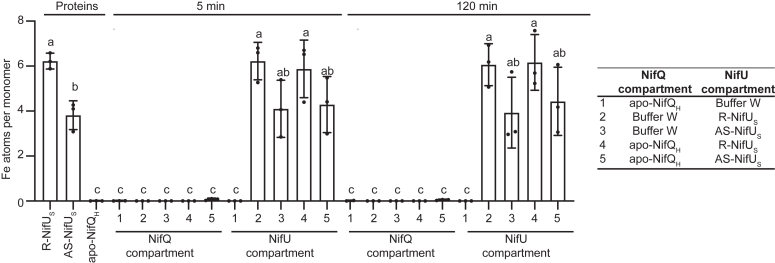
**NifQ requires physical interaction from NifU to receive iron.** Iron content per monomer of pure isolated proteins or from proteins separated by a 2-kDa pore-size cutoff dialysis membrane after five or 120 min incubation. Bars represent average iron content ± SD (n = 3). Different letters indicate statistically significant differences after being compared by ANOVA followed by Bonferroni’s multiple comparison test (*p* < 0.01). The dots represent each individual data point.

Iron binding to apo-NifQ could be due to sequestering the iron that may dissociate from NifU, instead of being the consequence of direct protein-protein transfer. If this were the case, separating the two proteins with a membrane that only allowed for iron diffusion but prevented the passage of the proteins, should still result in iron binding to apo-NifQ. However, when this control was carried out, using R-NifU_S_ or AS-NifU_S_, no iron was detected in the compartment containing apo-NifQ_H_ even when 120 min was allowed for iron to dissociate and diffuse (Fig. 5).

### NifQ primarily receives iron from NifU *in vivo*

**Figure 6:**
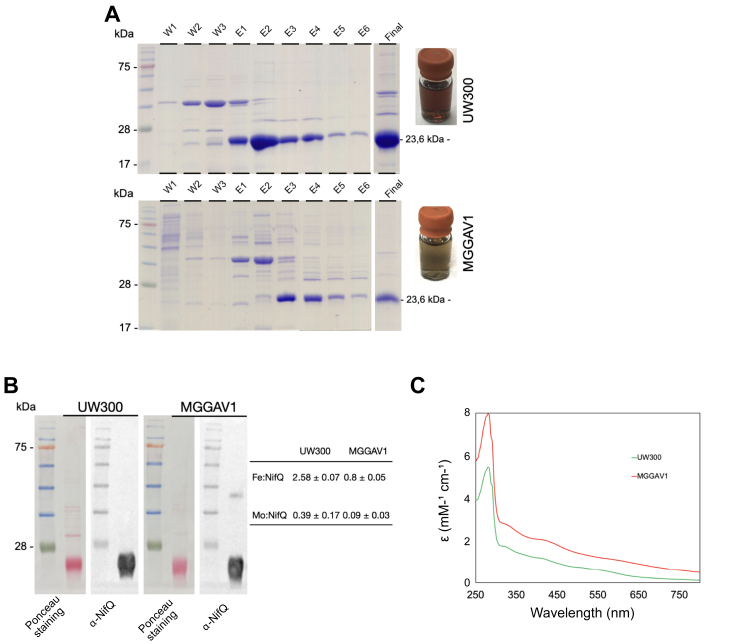
**NifU is the primary donor of [Fe-S] clusters to NifQ *in vivo*.***A*, His-NifQ purification using *A. vinelandii* UW3000 (wild type strain) and MGGAV1 (*ΔnifU* strain) cells. Elutions were tested for _H_NifQ presence Coomassie stained SDS gels. W1 to W3 indicates the three washes, and E1 to E6 the elution fractions, Final is the concentrated protein combined from E1 to E6. *B*, Ponceau staining and immunoblot of the purified concentrated _H_NifQ obtained from UW3000 or MGGAV1. The table shows the iron or molybdenum molar ratios of the purified NifQ_S_. *C*, visible molar extinction coefficients of pure NifQ isolated from UW3000 (*green*) or MGGAV1 (*red*).

In contrast to NifU, which is specific to nitrogenase, the *A. vinelandii* IscU protein has a more general role in providing clusters to enzymes involved in general metabolism (18). However, IscU can partially replace NifU when the latter is lost, as indicated by the partial recovery of the diazotrophic growth of a Δ*nifU* strain (19). Co-purification assays between reconstituted, strep-tagged IscU (R-_S_IscU) and apo-NifQ_H_ indicated that both proteins interact (Fig. S3), suggesting that IscU could replace NifU in [Fe-S] cluster delivery to NifQ. To test this hypothesis under *in vivo* conditions, expression of His_9_-tagged *nifQ* under a *nifH* promoter was compared in *A. vinelandii* wild-type and Δ*nifU* strains. While a similar yield of pure NifQ was obtained from both strains (113 and 165 mg/kg of cells) (Fig. 6*A*), the NifQ originating from the strain containing NifU had three times more iron (2.58 ± 0.07 Fe/monomer) than the one obtained from the Δ*nifU* background (0.8 ± 0.05 Fe/monomer) (Fig. 6*B*). Molybdenum levels were 0.39 ± 0.17 for the NifQ obtained from wild-type *A*. *vinelanddi* and 0.09 ± 0.03 for the one originating from the *nifU* mutant. Moreover, these proteins had different spectrophotometric properties, as indicated by their different molar extinction coefficients (Fig. 6*C*), which here were higher for the NifQ isolated from the *nifU* knock-out strain.

### NifU transfers a [Fe_4_-S_4_] cluster to NifQ

**Figure 7:**
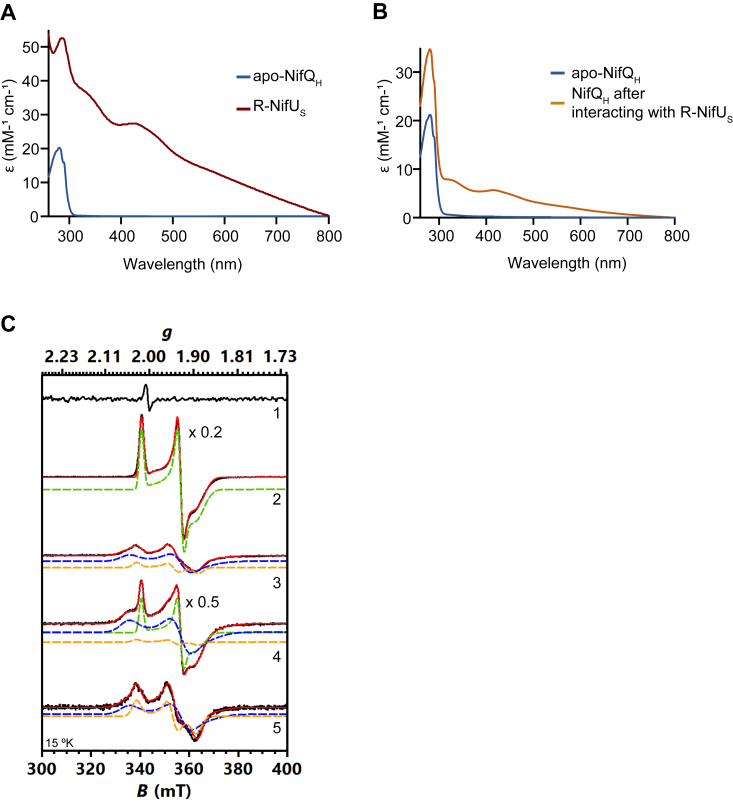
**NifQ receives a [Fe**_**4**_**-S**_**4**_**] cluster from NifU.***A*, Molar extinction coefficients in UV-Visible spectra. *B*, Molar extinction coefficients in UV-Visible spectra of flowthrough fraction after 5-min apo-NifQ_H_/R-NifU_S_ interaction. *C*, X-band cw-EPR spectra in the field range of 300 to 400 mT at 15 K under power-unsaturated conditions of the following NifQ and NifU samples: 1) apo-NifQ_H_, 2) R-NifU_S_, 3) flowthrough fraction after 5 min interaction of apo-NifQ_H_ and R-NifU_S_, 4) a second biological replicate of the spectrum of flowthrough fraction after 5 min interaction of apo-NifQ_H_ and R-NifU_S_, and 5) AS-NifQ_H_. *Black* lines present experimental spectra, *red* lines present total simulation, while colored dashed lines present individual simulation components: Species I (*green*), Species II (*blue*), and Species III (*orange*).

The presence of [Fe-S] clusters in a protein affects its UV-visible signature. R-NifU_S_ presented UV-vis absorption spectra characteristic of carrying O_2_-sensitive [Fe-S] clusters, with a peak around 330 nm and another around 420 nm (Fig. 7*A*). These peaks were not observed in apo-NifQ_H_ UV-vis absorption spectra, indicating the absence of any [Fe-S] cluster. Fractions obtained after the apo-NifQ_H_/R-NifU_S_ interaction were analyzed to determine their UV-vis absorption spectra. As shown in Fig. 7*B*, the absorption spectra from the flowthrough fraction, where only NifQ_H_ was detected by Western blot (Fig. 4*A*), presented the typical shoulder around 400 nm related to [Fe_4_-S_4_] cluster (8). To confirm the transfer of the [Fe_4_-S_4_] cluster to NifQ, continuous-wave electron paramagnetic resonance (cw-EPR) spectroscopy was performed in apo-, and AS-NifQ_H_, R-NifU_S_, and the flowthrough fractions resulting from the interaction between R-NifU_S_ and apo-NifQ_H_ which largely contains NifQ_H_ (Fig. 4*A*). The resulting spectra exhibited EPR g resonances that indicated the presence of three different species (Figs. 7*C*, S4*A*, Table 2). Spectral simulations revealed that Specie I (g = [2.02, 1.93, 1.89]) would represent the [Fe_2_-S_2_] present at the core region of NifU, as the [Fe_4_-S_4_] in NifU is known to be unstable and cannot be detected by EPR under dithionite reducing conditions (8). Temperature-dependent electronic relaxation of this EPR signal also supports the behavior of a [Fe_2_-S_2_] cluster (Fig. S4*B*) (20), where the signal did not show any intensity and linewidth change up to ∼ 50 K and only showed minor linewidth broadening up to ∼ 80 K after the signal was adjusted by temperature (signal x T, the Curie’s Law). The [Fe_4_-S_4_] is represented by Species II with g = [2.05, 1.93, 1.88], since it can be detected in AS-NifQ_H_ as well, and its signal disappeared when temperature increased to ∼ 50 K due to fast electronic relaxation (Fig. S4, *C* and *D*) (20). Species III (g = [2.03, 1.94, 1.89]) also exhibited EPR spectral characteristics compatible with a [Fe_2_-S_2_] cluster due to the temperature-dependent electronic relaxation behavior (Fig. S4, *C* and *D*), but slightly different from the ones observed in NifU, and with broader spectral lines, which could indicate heterogeneous artifacts or degradation product of species II. The EPR spectra for the two biological repetitions of flowthrough fractions containing NifQ_H_ show a profile largely compatible with AS-NifQ_H_, indicating the acquisition of a [Fe_4_-S_4_] cluster from R-NifU_S_. One of these replicates, however, had R-NifU_S_ contamination carry-over as indicated by the detection of Species I in the EPR, although at very low levels since it was not detected by Western blot (Fig. 4*A*).

**Table 2:** Spin concentration of the EPR observed species

Sample	Species I (spin/monomer)	Species II (spin/monomer)	Species III (spin/monomer)
apo-NifQ_H_	-	-	-
R-NifU_S_	0.527	-	-
Elution R-NifU_S_ + apo-_S_NifQ_H_ (rep. 1)	-	0.053	0.016
Elution R-NifU_S_ + apo-_S_NifQ_H_ (rep. 2)	0.120	0.200	0.016
AS-NifQ_H_	-	0.076	0.051

## Discussion

[Fe-S] proteins are present in all three domains of life, participating in a wide array of physiological processes that include DNA metabolism, energy transduction, or metabolic pathways (21). It has been estimated that 1 to 5% of bacterial proteins contain some [Fe-S] cluster (22). These prosthetic groups can be assembled on proteins *in vitro* by simply providing iron and sulfur, while *in vivo* they are synthesized over protein scaffolds in processes often requiring multiple enzymes and chaperones (21). The importance of these scaffold proteins is evidenced by their essential nature for cell metabolism and their specialization for different metabolic processes. Furthermore, to date, there is no known *de novo* synthesis of [Fe-S] clusters directly onto target apo-proteins. Consequently, a “bucket-brigade” of proteins direct the newly produced [Fe-S] clusters to the acceptor proteins (5, 21, 23). In this context, it is worth noting that in the model diazotroph *A. vinelandii* NifU is used as the primary scaffold for [Fe-S] cluster biosynthesis and transfer to certain nitrogenase components (7, 10), while IscU is used for more general-purpose proteins (19).

NifQ requires a pre-existing [Fe_3_-S_4_]^+^ cluster to participate as a molybdenum donor for FeMo-co biosynthesis (15, 16). The interaction observed in this work between NifU and NifQ suggests that NifU could be the source of a [Fe_4_-S_4_] precursor in NifQ. This is further supported by the increased iron content of NifQ after incubation with NifU and the spectroscopic signatures of the repurified NifQ, which presents the absorbance pattern of [Fe_4_-S_4_] groups characterized by a pronounced shoulder around 400 nm (8) and the EPR signals of a [Fe_4_-S_4_]. Interestingly, while IscU can interact with NifQ *in vitro*, in the absence of NifU *in vivo*, it is just able to transfer a limited amount of iron to NifQ. Furthermore, the composition of the NifQ clusters obtained from NifU or from IscU seems to differ as indicated by their different molar extinction coefficients.

The [Fe_4_-S_4_] cluster of NifQ would then have to lose one iron atom to generate the [Fe_3_-S_4_]^+^ cluster that has been observed to accommodate a molybdenum atom forming a [Mo-Fe_3_-S_4_] cluster (15). It is to be expected that additional proteins will establish complexes with NifQ to mediate molybdenum transfer and inclusion into this [Fe_3_-S_4_]^+^ cluster.

Functional interaction between two proteins can sometimes evolve into one single protein with two different domains, each one of them corresponding to one of the original enzymes. This is advantageous to channel the product of one enzyme to the next, reducing diffusion times, increasing local substrate concentrations, and improving kinetics overall (24). *A. vinelandii* NifU is an example of this domain evolution since it combines an N-terminal IscU scaffold motive, a central ferredoxin fold, and a C-terminal NfuA-like domain (7). Another protein essential for nitrogenase maturation, NifB can also be found as a standalone radical *S*-adenosylmethionine (SAM) domain that then interacts with the NifB-cofactor carrier NifX, or as a combination of both proteins in a single polypeptide (25). Consistent with an adaptation to optimize protein-protein interactions, some delta-proteobacteria (such as *Geoalkalibacter ferrihydricus* or *Malonomonas rubra*) contain NifQ as the C-terminal domain of a larger protein that also includes an N-terminal domain with high homology to IscU/NifU proteins. Interestingly, the additional domain only shares homology with the N-terminal region of *A. vinelandii* NifU, which could indicate that the [Fe_4_-S_4_] clusters transferred to NifQ would mainly be synthesized in N-terminal NifU.

The interaction between two proteins exchanging substrates must be relatively fast and labile to work at optimal conditions and limit the subset of proteins and substrates lost in unproductive interactions. For instance, Cu^+^-chaperone CopZ rapidly dissociates from Cu^+^-transporting ATPase CopA after transferring Cu^+^ to prevent the apo-chaperone from blocking the transfer site (26, 27). NifQ-NifU interaction is similarly conditional to the metalation state of NifQ. When NifQ already contains an [Fe-S] cluster -the holo-NifQ used in this study-the interaction with NifU does not occur or is severely weakened.

Beyond the specific metalation of NifQ, its interaction with NifU and the likely dependency on NifU activity signals the position in which iron and molybdenum metabolism for biological nitrogen fixation are coordinated. Only when sufficient iron is allocated for NifU, molybdenum might be used for NifQ. This co-regulation of both elements is also present in other molybdenum-dependent reactions, such as the synthesis of the molybdopterin-based molybdenum cofactor (28).

**Figure 8:**
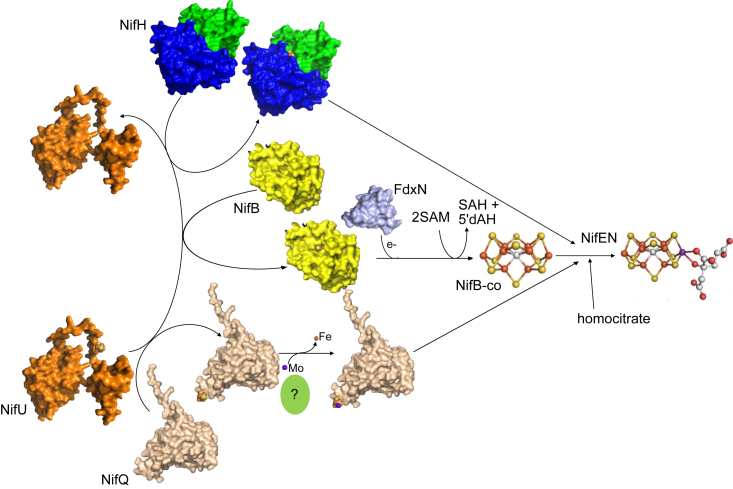
**NifU provides [Fe**_**4**_**-S**_**4**_**] clusters to NifH, NifB, and NifQ for FeMo-co biosynthesis.** [Fe_4_-S_4_]-containing NifU transfers this group to NifH, NifB, and NifQ. The latter likely interacts with an unknown protein that would mediate the substitution of a Fe for a Mo in the cluster. This group will be transferred together with the NifB-co synthesized by NifB with electrons likely provided by FdxN, and homocitrate to NifEN, where in a process assisted by NifH, FeMo-co will be produced. Structural models for NifU, NifQ, and FdxN were generated using AlphaFold (29) and the Protein Data Bank accession 1CP2 for NifH and 6Y1X for NifB. All structures were visualized with PyMOL (Schörindger, Inc.).

In summary, NifU transfers [Fe_4_-S_4_] clusters to at least three major sets of [Fe-S] proteins involved in FeMo-co synthesis: NifH, NifB, and NifQ (Fig. 8). This information is relevant as nitrogenase elements are being introduced and expressed in plants for the development nitrogen-fixing crop plants. Co-expression with NifU has already been shown to be essential for NifH activity when purified from plant chloroplasts (30), as well as for NifB obtained from yeast mitochondria (17). Therefore, it should be expected that similar co-expression with NifU would be needed for a functional NifQ-mediated molybdenum delivery pathway to nitrogenase in plants. Moreover, it remains yet to be solved how NifDK obtains the precursor [Fe_4_-S_4_] groups for P-cluster biosynthesis. Based on these results a direct transfer from NifU or one mediated by NifH could be hypothesized.

## Experimental procedures

### *E. coli* and *A. vinelandii* strains and plasmids

*E. coli* strain BL21 (DE3) was used to express the proteins used in this study. The plasmid pN2LP30 was used to produce untagged NifU and NifS in *E. coli* (Table S1). This plasmid was obtained by amplifying the *A. vinelandii nifUS* genes with primers 2495 and 2496 (Table S2) and using ELIC cloning (31) to introduce them in the *Nco*I/*Not*I digested pRSF*iscmetK*Duet-1 plasmid. To generate a NifQ_H_ expressing vector, the primers 1184 and 1185 (Table S2) were used to amplify the *nifQ* sequence from the *A. vinelandii* genomic DNA. The resulting amplicon was digested with *Pst*I and *Not*I and cloned into previously *Pst*I/*Not*I-digested pTrc99 A (Table S1). To produce _S_NifQ, the amplicon obtained from *A. vinelandii* genomic DNA using the primers NifQ-5′ and NifQ-3’ (Table S2) was digested with *Nde*I and *BamH*I and cloned in a similarly digested pT7-7 vector. Strep-tag was added to this vector by ligating at the *Nde*I site the overlapping oligonucleotides *Nde*I-Strep-tag-5′ and *Nde*I-Strep-tag-3’ (Table S2). The same procedure was used to fuse the Strep-tag to *nifS*-expressing vector pDB21223 (9) (Table S1). _H_NifU was obtained from cells transformed with plasmid pRHB609 (32). To produce NifU_S_, a 174 DNA fragment containing the last 99 nucleotides of *NifU* fused to the Strep-tag was synthesized (Integrated DNA Technologies, Coralville, IA), digested with *Sac*I and *BamH*I, and ligated in similarly digested *NifU*-encoding plasmid pDB525 (7) (Table S1). IscU_S_ was obtained from *A. vinelandii* genomic DNA using the primers 5NcoI-IscU-CStrep and 3NdeI-IscU-CStrep (Table S2). This amplicon was digested with *NcoI* and *NdeI* restriction enzymes and cloned in pET16bStrep plasmid (Table S1), previously digested with the same restriction enzymes, by ligation.

*A. vinelandii* strain UW300 (P_*nifH*_::*his*_9_-*nifQ*) was used to purify His-NifQ known to present a [Fe_3_-Mo-S_4_] cluster (15). Additionally, *A. vinelandii* strain MGGAV1 was engineered to purify His-NifQ in a *ΔnifU* background. For this purpose, Dennis Dean at Virginia Tech kindly provided the *A. vinelandii* strain DJ105 that presents an in-frame *nifU* deletion (33). Using plasmid pRHB272 (Table S1), it was possible to integrate His-*nifQ* under the *nifH* promoter in DJ105 using ampicillin selection.

### Culture conditions for nif protein expression

In general, expression of cloned *nif* genes in *E. coli* was induced with 1 mM isopropyl β-D-thiogalactoside (IPTG) in cells growing in LB media supplemented with 100 μg/ml ampicillin at OD_600_ ≈ 0.6. After 3 h of induction at 37 °C, cells were collected by centrifugation at 4000*g* for 10 min. Cells producing NifU_S_ were grown in LB media supplemented with 100 μg/ml ampicillin, with 0.2 mM ferric ammonium citrate and 2 mM L-cysteine. Induction started at OD_600_ ≈ 0.6 by adding 0.5 mM IPTG and lasted 5 to 6 h at 37 °C. _H_NifU induction was performed at OD_600_ ≈ 0.7 with 1 mM IPTG and 0.1 mg/l Fe(NH_4_)_2_(SO_4_)_2_ for 14 h at 18 °C and 150 rpm. IscUs was induced with 1 mM IPTG, 2 mM L-cysteine, and 0.2 mM ferric ammonium citrate for 3 h at 30 °C and 105 rpm.

*A. vinelandii* cells for protein purification were cultivated in a 300 l fermentor (Bioprocess Bioengineering) in 150 l batches of modified Burk’s medium supplemented with ammonium acetate (2.8 mM). Nitrogenase derepression was initiated when cultures reached a 4.5 optical density (600 nm). After this, the culture was concentrated down to 10 to 15 l using a hollow fiber system (GE Healthcare), and the cell soup was removed from the fermenter. Fresh Burk media was made in the reactor without ammonium acetate, adding the cell soup was added, and finally, cells were derepressed for an additional 4 h. Cells were immediately collected and stored at −80 °C.

### Protein purification from *E. coli* cells

Strep-tagged proteins were purified by Strep-Tactin XT affinity chromatography (SATC). Approximately 15 to 20 g of recombinant *E. coli* BL21(DE3) cells were resuspended for 30 min in 80 ml of lysis buffer A (50 mM Tris-HCl pH 8.0, 100 mM NaCl and 10% glycerol, 1 mM phenylmethylsulfonyl fluoride (PMSF)). Cells were lysed in a French Press cell at 1500 lb per square inch. The cell-free extract (CFE) was obtained after removing cell debris by centrifugation at 63,000*g* for 1 h at 4 °C and filtration with 0.45 μm pore size syringe filters (Sartorius). CFE was loaded onto a 1 ml Gravity flow Streptactin-XT high-capacity column (IBA Lifesciences), previously equilibrated with buffer A. The column was then washed 5 times with two column volumes (CV) of buffer A per wash. Bound protein was eluted in three steps with 1, four and 2 CV of buffer A containing 50 mM biotin per step.

His-tagged proteins were purified by Ni-NTA affinity chromatography. Approximately 20 to 25 g of recombinant *E. coli* BL21(DE3) cells were resuspended for 30 min in 100 ml of lysis buffer W (100 mM Tris-HCl pH 8.0, 150 mM NaCl, and 10% glycerol) supplemented with 1 mM PMSF. Cells were lysed and CFE was obtained as described above. CFE was loaded onto a 2 ml Ni-NTA Agarose column (Qiagen) equilibrated with buffer W supplemented with 5 mM imidazole. Column was washed 6 times with 1 CV of buffer W with 5 mM imidazole and 6 times with 1 CV of buffer W with 20 mM imidazole per wash. Protein was eluted in two batches with buffer W containing 150 and 300 mM imidazole, respectively.

Apo-NifQ purifications were carried out in aerobic conditions to promote the destruction of [Fe-S] clusters and the elimination of bound iron. All other proteins were purified under anaerobic conditions (<5.0 ppm O_2_) inside a glovebox (COY Laboratories) using buffers previously made anaerobic by sparging with N_2_ overnight. Purification fractions were analyzed by electrophoresis.

Elution fractions were concentrated with 10 kDa cut-off pore-size centrifugal membrane devices (Amicon Ultra-15, Millipore). Centrifugation procedure was performed at 4000 x *g* for 45 min and this step was repeated until estimated biotin or imidazole concentration was lower than 50 nM and 500 nM, respectively. Purified proteins were frozen and stored in liquid N_2_.

### Protein Purification from *A. vinelandii* cells

_H_NifQ purifications from UW300 and MGGAV1 cells were carried out simultaneously. For this purpose, twin glovebox setups equipped with GE AKTA Prime Plus purifiers were utilized (GE Healthcare). Purifications were performed at 14 °C as follows: 115 g of cells were resuspended in the same volume of buffer B (50 mM Tris-HCl pH 7.9, 150 mM NaCl) supplemented with PMFS and DNAse I inside a CoyLabs glovebox (CoyLabs). The cell soup was lysed applying a 1200 bar pressure using an IKA HPH 2000/4 DH homogenizer (IKA). A cell-free extract was obtained after applying 69,000*g* for 2 hours. Cell-free extracts were loaded in 5 ml pre-packed Ni^2+^-IMAC columns (Cytiva) previously equilibrated in buffer B. Loaded columns were washed using 20 CV of buffer B and an additional 20 CV of buffer B supplemented to 25 mM imidazole. Protein was eluted applying a 0 to 300 mM imidazole gradient in 20 ml of buffer B. NifQ-rich elutions, selected after Coomassie-stained SDS-gel analysis, were concentrated using 10 kDa centricons (Millipore), and desalted using a Hiprep 26/10 column (Cytiva) previously equilibrated in buffer B. Purified _H_NifQ was stored as pellets in liquid N_2_. Protein and iron content was determined using BCA (Pierce, IL, USA) and bipyridyl method (34), respectively.

### *In vitro* [Fe-S] cluster reconstitution

Strep-tagged or His-tagged NifU purified from *E. coli* were reconstituted *in vitro* as described (35) with slight modifications. 20 μM of NifU dimer was prepared in 100 mM MOPS (pH 7.5) buffer containing 8 mM 1,4-dithiothreitol (DTT) and incubated at 37 °C for 30 min. To this mixture, 1 mM L-cysteine, 1 mM DTT, 225 nM NifS, and 0.3 mM (NH_4_)_2_Fe(SO_4_)_2_ were added. Iron additions were divided in three steps of 15 min each until reaching the final concentration of 0.3 mM. The reconstitution mixture was kept in ice for 3 h and then desalted using 10-kDa cutoff pore size centrifugal membrane devices (Amicon, Millipore) to remove excess reagents. R-NifU protein was stored in liquid N_2_ until use. IscU reconstitution was carried out with 20 mM of purified protein in 100 mM MOPS pH 7.5, 9 mM DTT, and incubated for 30 min at room temperature. Then, 500 nM of NifS, 1 mM L-cysteine, and 0.4 mM (NH_4_)_2_Fe(SO_4_)_2_ were added. Iron addition was done in 10 successive steps with 15 min in between to avoid precipitation. Samples were incubated for 4 h at room temperature, and unbound iron was removed by desalting with 10-kDa cutoff pore size centrifugal membrane devices.

### Protein-protein interaction assays

Interaction assays were carried out for 5 min unless otherwise stated using 10 nmol of each protein in a glovebox (COY Laboratories) under anaerobic conditions. Those involving apo-_S_NifQ took place in buffer A. _S_NifQ and its interacting proteins were recovered passing the solution through a 200 μl Gravity flow Streptactin-XT column (IBA Lifesciences), previously equilibrated with anaerobic buffer A. Column was washed 5 times with 2 CV of buffer A. The elution of target proteins from the resin was carried out by applying 0.5 CV, 1.4 CV and 0.8 CV of 2.5 mM desthiobiotin in buffer A.

When using NifQ_H_ as bait, the interaction was carried out in buffer W. Proteins were separated using a 200 μl Ni-NTA agarose column equilibrated with anaerobic 5 mM imidazole in buffer W. The column was washed 6 times with 2 CV of 5 mM imidazole in buffer W and 6 times with 2 CV of 20 mM imidazole in buffer W per wash. Elution was performed with 150 mM imidazole in buffer W. To determine iron transfer from R-NifU_S_ to apo-NifQ_H_, 40 nmol of each protein was incubated from 5 min at room temperature and passed through a Streptactin-XT high-capacity column previously equilibrated in buffer W. Column was washed seven times with 5 CV of buffer W and eluted with 4 CV of 50 mM biotin in buffer W.

To assess the interaction between holo-NifQ_H_ and AS-NifU_S_, 10 nmol of AS-_S_NifU were immobilized on a 200 μl Gravity flow Streptactin-XT column, previously equilibrated with anaerobic buffer A. Column was washed twice with 2 CV of buffer A and 10 nmol of holo-NifQ_H_ were loaded onto the AS-_S_NifU-charged column. This column was washed 3 times with 3 CVs of buffer and eluted with 50 mM biotin in buffer A. The interaction between R-IscU_S_ and apo-NifQ_H_ was similarly carried out.

To determine iron transfer, UV-Vis spectra, and EPR analyses, 50 nmol of apo-NifQ_H_ and 50 nmol of R-NifU_S_ were incubated in a glovebox for 5 min at room temperature. Proteins were separated by passing through a 1 ml Strep-tactin XT 4Flow High-capacity column (IBA, Göttingen, Germany) previously equilibrated in buffer W. The column was washed with seven CVs of buffer W, and the proteins were eluted with 50 mM biotin in Buffer W. To test the diffusion of iron from R-NifU_S_ to apo-NifQ_H_, 50 nmol of each protein were incubated for 5 and 120 min inside an anaerobic glovebox (COY Laboratories), separated by inserting a 2 kDa pore-size cutoff dialysis membrane, previously equilibrated for 1 h with buffer W. Controls with only apo-NifQ_H_ on R-NifU_S_, AS-NifU_S_ on the other side of the membrane were carried out at the same time. At the indicated times, samples from both membrane sides were collected to determine the protein and iron concentration.

Protein content in all selected fractions was analyzed by SDS-PAGE using 12% acrylamide/bisacrylamide (37.5:1) gels and visualized by Coomassie Brilliant Blue staining (36). For immunoblot analysis, proteins were transferred to nitrocellulose membranes for 45 min at 20 V using a Transfer-Blot Semi Dry system (Bio-Rad). Immunoblot analyses were carried out with antibodies raised against *A. vinelandii* NifQ (1:2500 dilution), NifU (1:2500 dilution) and NifS (1:1500 dilution) (32, 37). A horseradish peroxidase-conjugated anti-rabbit antibody (Invitrogen) diluted 1:15,000 was used as a secondary antibody. Chemiluminescent detection was carried out according to Pierce ECL Western Blotting Substrate kit's instructions (ThermoFisher Scientific) and developed in an iBright FL1000 Imaging System (ThermoFisher Scientific). Protein content was determined using BCA (Pierce) and iron content with Atomic Absoption Spectroscopy.

### Atomic absorption spectroscopy

Samples were mineralized in 37.5% analytic grade nitric acid for 10 min at 80 °C. Samples were then diluted to a total concentration of 2% nitric acid. Iron and molybdenum concentrations were determined in an Atomic Absorption Spectrometre ContrAA 800G (AnalytikJena, Jena, Germany) using commercially available analytic grade metal standards (Inorganic Ventures).

### Ultraviolet-visible spectroscopy

UV-visible absorption spectra were collected under anaerobic conditions (<0.1 ppm O_2_) inside a glovebox (MBraun) in septum-sealed cuvettes to avoid the O_2_ contamination during the measurements in the Shimadzu UV-2600 spectrophotometer. Absorption (225 nm to 800 nm) was recorded.

### Electron paramagnetic resonance (EPR) spectroscopy

Protein samples were prepared in 100 mM Tris-HCl pH 8.0, 350 mM NaCl, 10% glycerol, 1 mM dithionite (DTH). X-band (9.64 GHz) cw-EPR spectra were recorded on a Bruker Elexsys spectrometer equipped with an Oxford ESR 910 cryostat and a Bruker bimodal cavity. The microwave frequency was calibrated with a frequency counter and the magnetic field with an NMR G m. The temperature was calibrated with a carbon-glass resistor temperature probe (CGR-1-1000; LakeShore Cryotronics) located into an EPR tube. For all EPR spectra, a modulation frequency and amplitude of 100 kHz and 1 mT were used. The first-derivative spectra were recorded at 1024 points with an integration time of 150 milliseconds. EPR spectral simulations were performed using the simulation software SpinCount (38). The spin was quantified by relative to a 1.2 mM Cu(II)ethylenediaminetetraacetic solution with 10% glycerol (v/v). Two EPR samples, independently prepared from two different NifQ and NifU interaction assays, were measured.

### Statistical methods

SPSS software (Statistical Package for Social Sciences) was used for statistical analyses. The data were compared using one-way analyses of variance (ANOVA) followed by Bonferroni’s multiple comparison test (*p* < 0.01).

## Data availability

The authors declare that the data supporting the findings of this study are available within the article, its supplementary information and data, and upon request.

## Supporting information

This article contains supporting information.

## Conflict of interest

The authors declare that they have no conflict of interest with the contents of this article.
