# Supplementary figures and images for: Azotobacter vinelandii scaffold protein NifU transfers iron to NifQ as part of the iron-molybdenum cofactor biosynthesis pathway for nitrogenase
Source: J Biol Chem. 2024 Oct 22;300(11):107900. doi: 10.1016/j.jbc.2024.107900 (PMC11605450; doi:10.1016/j.jbc.2024.107900)

**FIGURE S1**

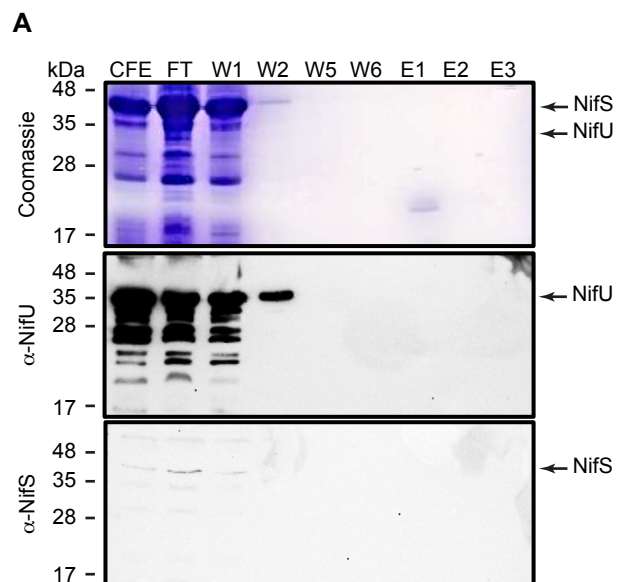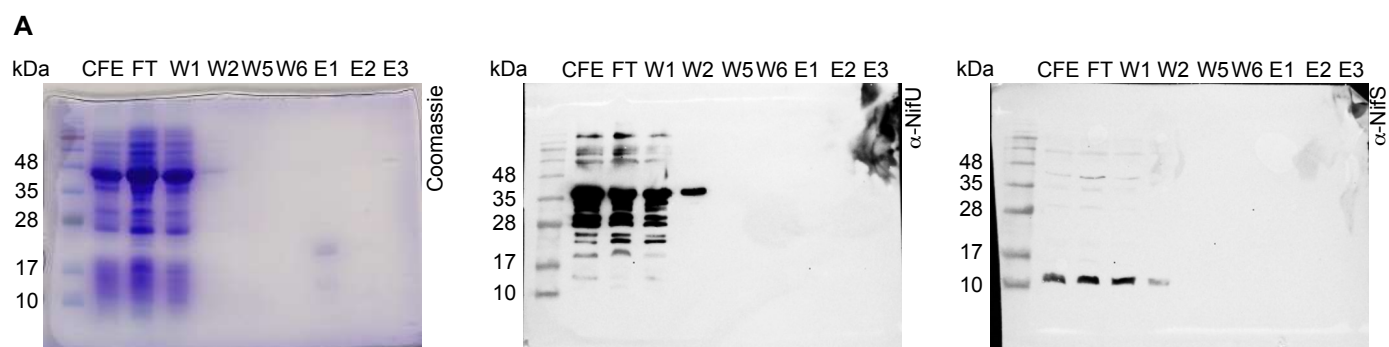

**FIGURE S2**

**A**

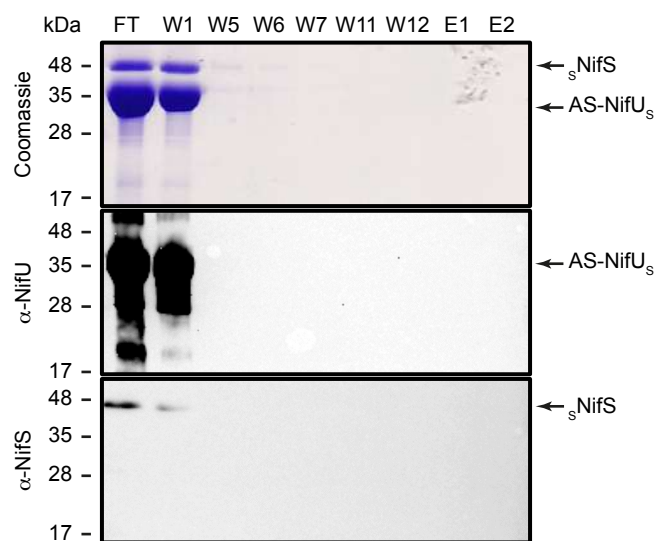

**B**

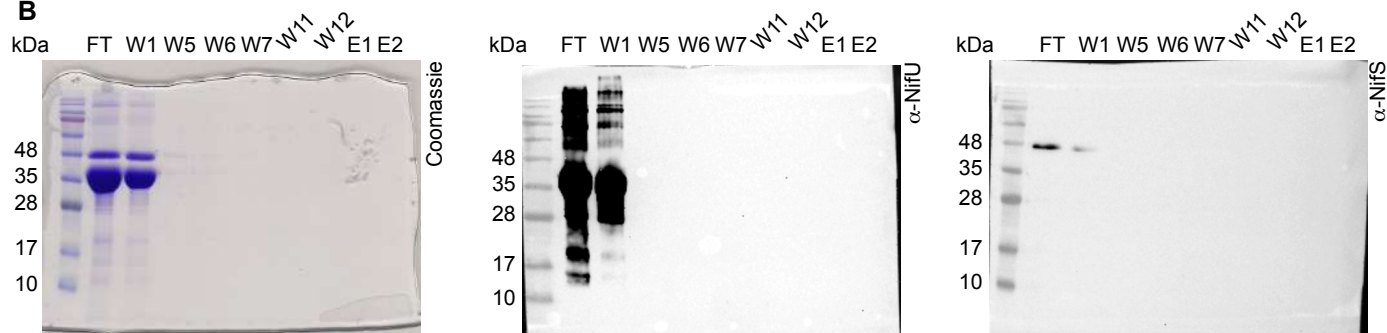

**FIGURE S3**

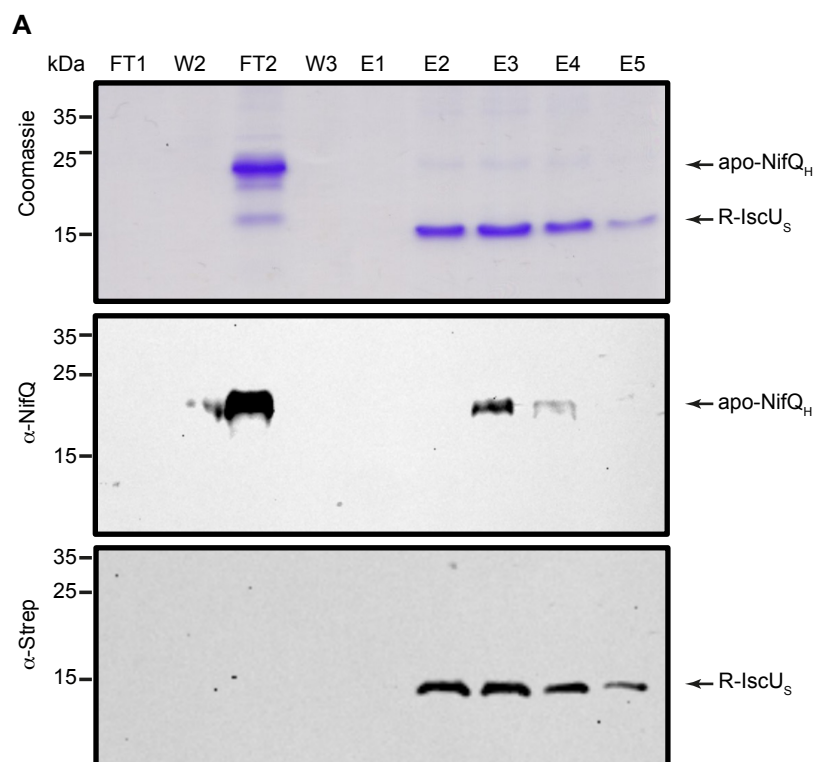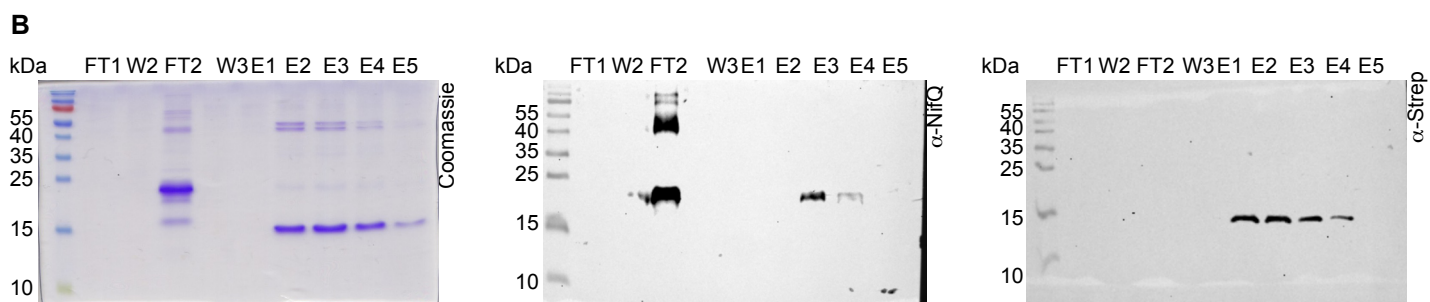

FIGURE S4

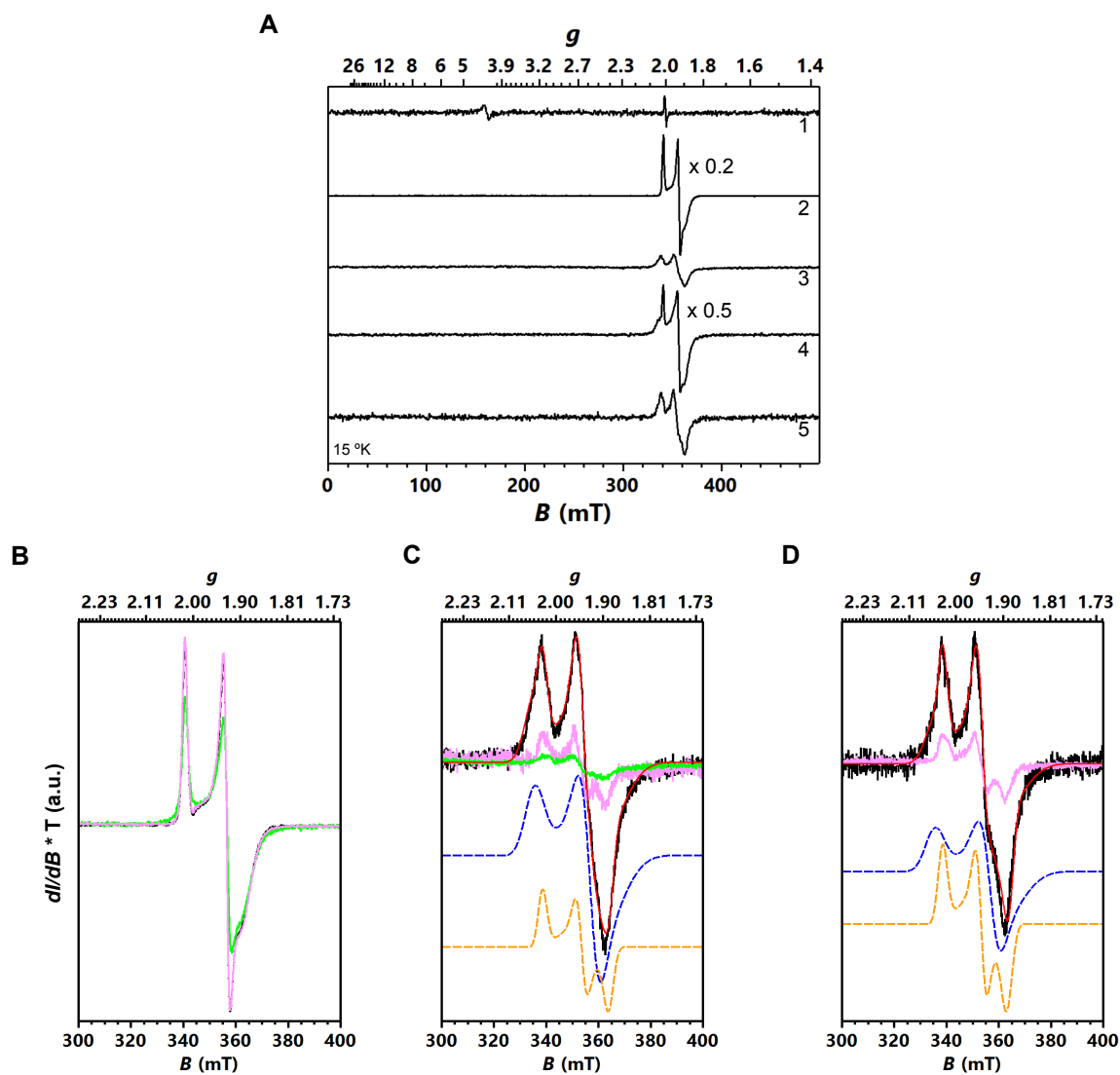

**FIGURE S5**

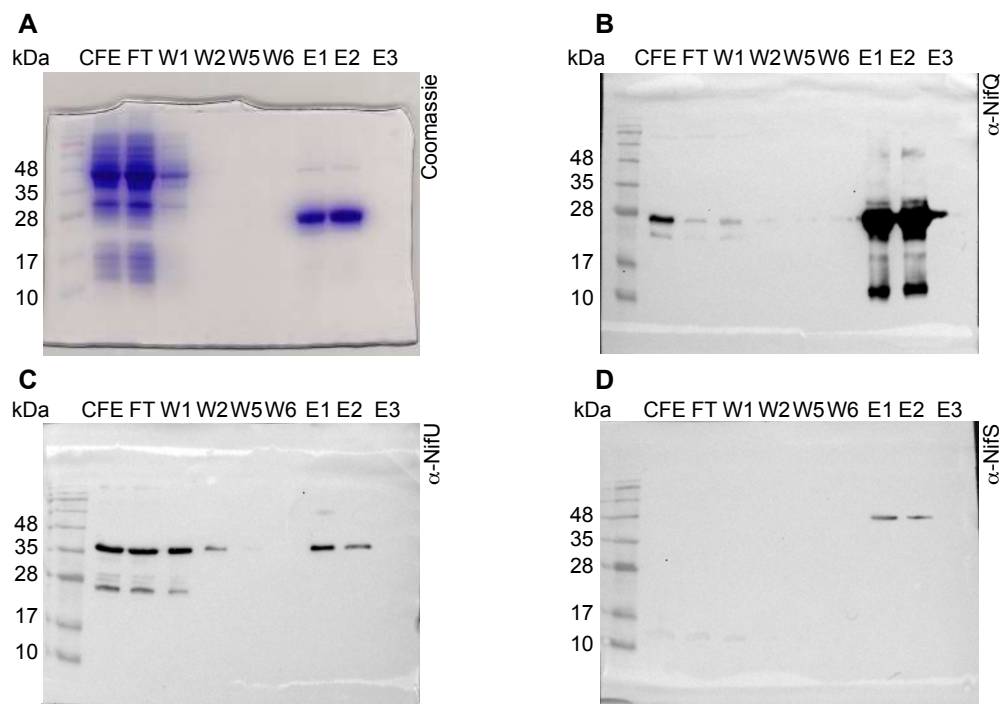

**FIGURE S6**

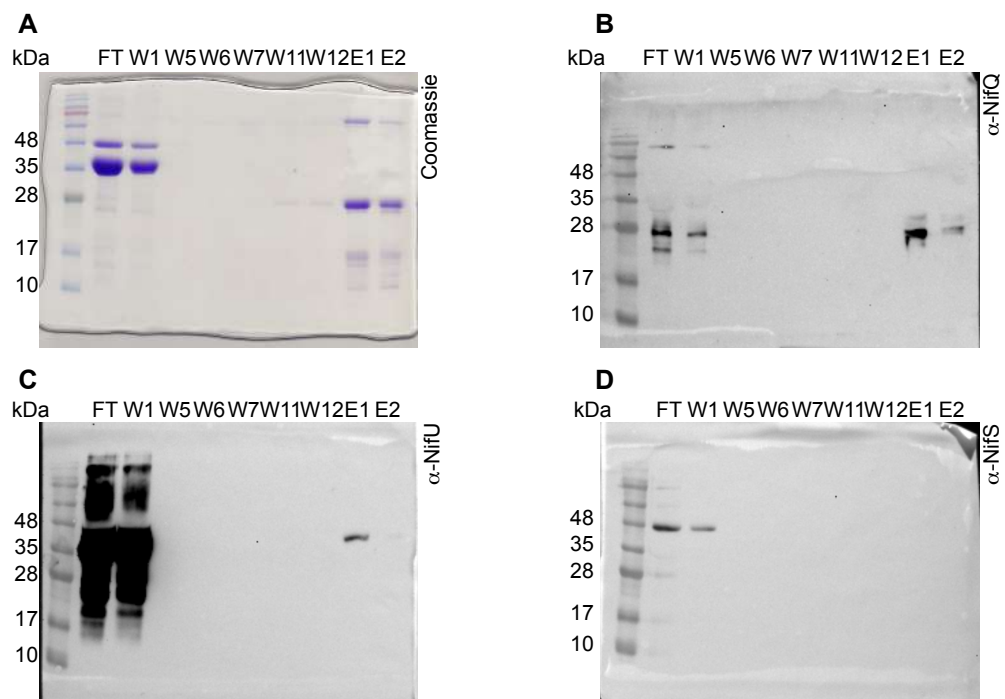

**FIGURE S7**

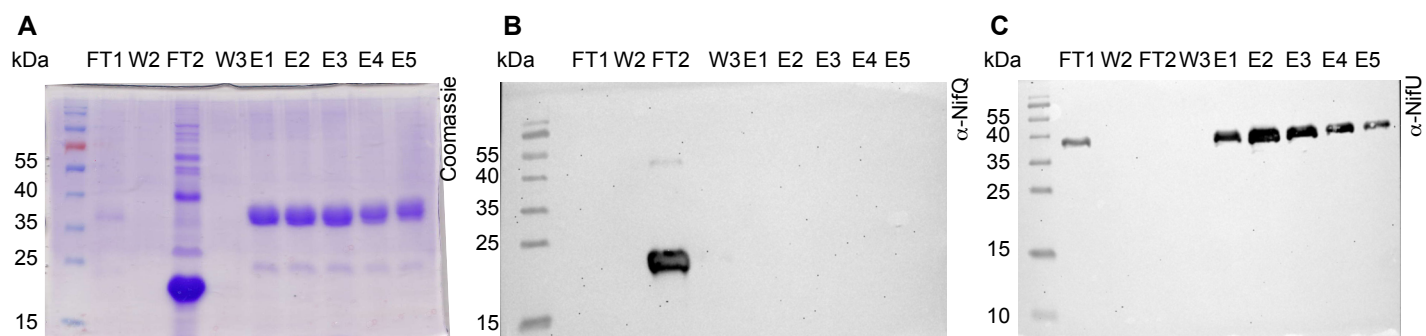

**FIGURE S8**

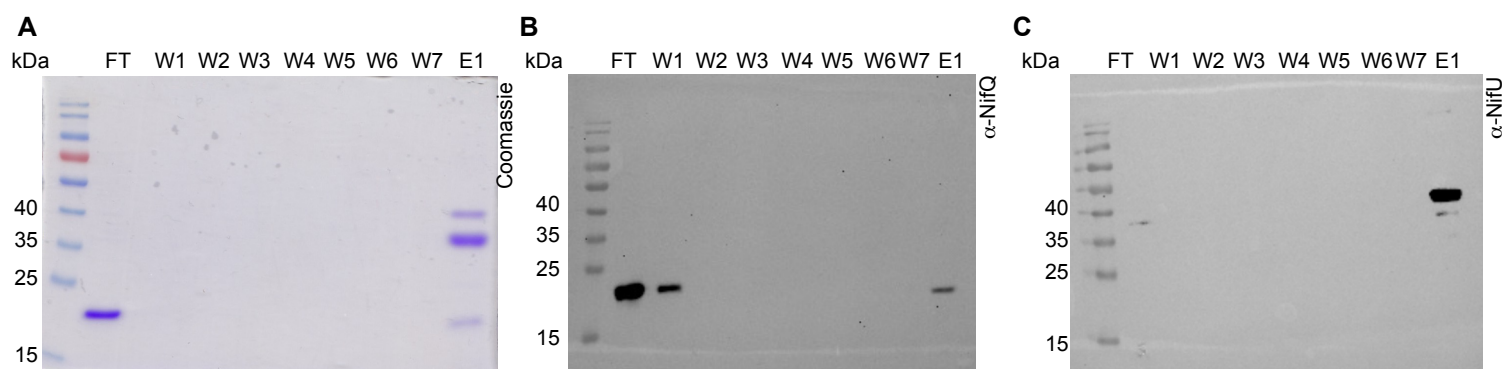

Supplement: Supplemental Figures [file mmc1.pdf]
