# Supplementary material for: Azotobacter vinelandii scaffold protein NifU transfers iron to NifQ as part of the iron-molybdenum cofactor biosynthesis pathway for nitrogenase
Source: J Biol Chem. 2024 Oct 22;300(11):107900. doi: 10.1016/j.jbc.2024.107900 (PMC11605450; doi:10.1016/j.jbc.2024.107900)
Supplement: Supplemental Figure and Table [file mmc2.pdf]

Figure S1. NifU and NifS do not bind to the Streptactin resin. A. Top panel shows the Coomassie staining of an SDS-PAGE of cell free extract (CFE), flowthrough (FT), wash (W1-W6) and elution (E1-E3) fractions of *nifU* and *nifS*-expressing *E. coli* extracts passed through a Streptactin column. The remaining panels are the immunoblots of the same fractions developed with anti-NifU or an anti-NifS antibodies. Images show a representative assay (*n*=2). **B.** Uncropped Coomassie-stained gels shown in panel A (left panel), uncropped immunoblots corresponding to an anti-NifU (centre panel), or an anti-NifS (right panel) antibodies.

Figure S2. AS-NifU$_S$ and $_S$NifS proteins do not bind to a Ni^{2+} column. A. Top panel shows the Coomassie staining of an SDS-PAGE of flowthrough (FT), wash (W1-W12) and elution (E1-E2) fractions of a mixture solution containing AS-NifU$_S$ and $_S$NifS passed through a Ni^{2+} column. The remaining panels are the immunoblots of the same fractions developed with anti-NifU or an anti-NifS antibodies. Images show a representative assay (*n*=3). **B.** Uncropped Coomassie-stained gel shown in panel A (left panel), uncropped immunoblots with an anti-NifU (centre panel), or an anti-NifS (right panel) antibodies.

Figure S3. **R-IscU$_S$ interacts with apo-NifQ$_H$**. **A.** Top panel shows the Coomassie staining of an SDS-PAGE of a mixture solution containing R-IscU$_S$, and apo-NifQ$_H$ passed through a Strep-column. FT1 is the flow-through fraction obtained after loading R-IscU$_S$ onto the Strep-column. W2 is the second wash fraction. FT2 is the flow-through fraction obtained after loading apo-NifQ$_H$ onto the column. W3 is the third wash fraction after passing apo-NifQ$_H$ over R-IscU$_S$ - charged column. E1, 2, 3, 4 and 5 are elution fractions. The remaining panels are the immunoblots of the same fractions developed with anti-NifQ, or anti-Strep antibodies. Images show a representative assay (n=3). **B.** Uncropped Coomassie-stained gels shown in panel A (left panel), uncropped immunoblots an anti-NifQ (centre panel), or an anti-NifU (right panel) antibodies.

Figure S4. Temperature dependent relaxation of X-EPR spectra. **(A)**. X-band cw-EPR spectra in the field range of 0-500 mT at 15 ºK under power-unsaturated conditions of the following NifQ and NifU samples: 1) apo-NifQ$_H$, 2) R-NifU$_S$, 3) flowthrough fraction after 5 min interaction of apo-NifQ$_H$ and R-NifU$_S$, 4) a second biological

replicate of the spectrum of flowthrough fraction after 5 min interaction of apo-NifQ$_H$ and R-NifU$_S$, and 5) AS-NifQ$_H$. Some of the spectra are scaled by the indicated factors. (**B**) Temperature dependent X-band cw-EPR spectra of R-NifU$_S$ in the field range of 300-400 mT under power unsaturated condition 15 ºK (black), 48 ºK (pink) and 83 ºK (green). (**C**) Temperature dependent X-band cw-EPR spectra of repetition 1 of the flowthrough fraction after 5 min interaction of apo-NifQ$_H$ and R-NifU$_S$ in the field range of 300-400 mT under power unsaturated condition 15 ºK (black), 48 ºK (pink) and 83 ºK (green). Red lines present total simulation of the data at 15 ºK, while colored dashed lines present individual simulation components: Species II (blue), and Species III (orange). (**D**) Temperature dependent X-band cw-EPR spectra of AS-NifQ$_H$ in the field range of 300-400 mT under power unsaturated condition 15 ºK (black), and 48 ºK (pink). Red lines present total simulation of the data at 15 ºK, while colored dashed lines present individual simulation components: Species II (blue), and Species III (orange). The spectra were scaled by temperature (first-derivative signal * T). a.u. are stands for arbitrary units.

Figure S5. Uncropped Coomassie-stained gels shown in Figure 1 (**A**). Uncropped immunoblots shown in Figure 1 that correspond to immunoblotting with an anti-NifQ antibody (**B**), an anti-NifU antibody (**C**) or an anti-NifS antibody (**D**).

Figure S6. Uncropped Coomassie-stained gels shown in Figure 2 (**A**). Uncropped immunoblots shown in Figure 2 that correspond to immunoblotting with an anti-NifQ (**B**), an anti-NifU (**C**), or an anti-NifS (**D**) antibodies.

Figure S7. Uncropped Coomassie-stained gels shown in Figure 3 (**A**). Uncropped immunoblots shown in Figure 3 that correspond immunoblotting with an anti-NifQ (**B**), or an anti-NifU (**C**) antibodies.

Figure S8. Uncropped Coomassie-stained gels shown in Figure 4 (**A**). Uncropped immunoblots shown in Figure 4A that correspond immunoblotting with an anti-NifQ (**B**), or an anti-NifU (**C**) antibodies.

Table S1: Plasmids used in this study.

Table S2: Primers used in this study.
