# Supplementary material for: Azotobacter vinelandii scaffold protein NifU transfers iron to NifQ as part of the iron-molybdenum cofactor biosynthesis pathway for nitrogenase
Source: J Biol Chem. 2024 Oct 22;300(11):107900. doi: 10.1016/j.jbc.2024.107900 (PMC11605450; doi:10.1016/j.jbc.2024.107900)
Supplement: Supplemental Table S1 [file mmc3.pdf]

Table S1. Plasmid used in this work

Plasmid	Used for	Source
pDB2123	To overexpress streptag-*nifS* under T7 promoter, Ap^R.	Dean laboratory collection
pDB2146	To overexpress streptag-*nifQ* under T7 promoter, Ap^R.	Dean laboratory collection
pDB2174	To overexpress *nifU*-streptag under T7 promoter, Ap^R.	Dean laboratory collection
pTrc99A	For overproduction of native proteins under pTrc promoter, Km^R	Pharmacia[a]
pN2EB18	To overexpress *nifQ*-6xHis under pTrc promoter, Km^R.	This work
pRHB609	To overexpress 6xHis-*nifQ* under T7 promoter, Ap^R.	30
pT7-7	For overproduction of native proteins under T7 promoter, Ap^R	10.1073/pnas.82.4.1074.
pN2LP30	To overexpress *nifUS* under T7 promoter, Ap^R.	Rubio laboratory collection
pET16bStrep	To overexpress *IscU*-streptag under T7 promoter, Ap^R.	Rubio laboratory collection
pRHB272	To overexpress *9x-NifQ* under nifH promoter, Ap^R.	15

Abbreviations:
Ap, ampicillin;
Km, kanamycin;
R, resistance.

[a]Amersharm Pharmacia Biotech, Uppsala, Sweden.
