# Supplementary material for: Azotobacter vinelandii scaffold protein NifU transfers iron to NifQ as part of the iron-molybdenum cofactor biosynthesis pathway for nitrogenase
Source: J Biol Chem. 2024 Oct 22;300(11):107900. doi: 10.1016/j.jbc.2024.107900 (PMC11605450; doi:10.1016/j.jbc.2024.107900)
Supplement: Supplemental Table S2 [file mmc4.pdf]

Table S2. Primers used in this study.

Name	Sequence	Use
2495	TTAATAAGGAGATATACCATGGCCTGGGATTATTCGGAAA	Cloning of *nifUS* in pN2LP30
2496	TTCGACTTAAGCATTATGCGGCCGCTCAGCCGTAGACCGG	Cloning of *nifUS* in pN2LP30
1184	AAATTCTGCAGATGGGCAGCGCCGCG	Generation of $NifQ_H$
1185	AATTGCGGCCGCGAGAATCGGGTCATATCTCTGCTCC	Generation of $NifQ_H$
nifQ-5’	CATG CAT ATG GGC AGC GCC GCG GCC	Amplification of NifQ
nifQ-3’	CTAC GGA TCC TGG CCG GCC AGC AGG	Amplification of NifQ
*Nde*I-Strep-tag -5'	TATGGCTAGCTGGAGCCACCCGCAGTTCGAAAAACA	Addition of Strep-tag to *Nde*I sites
*Nde*I-Strep-tag -3'	TATGTTTTTCGAACTGCGGGTGGCTCCAGCTAGCCA	Addition of Strep-tag to *Nde*I sites
5NcoI-IscU-CStrep	AGGCCATGGCTTACAGTGACAAGGTCATCG	Cloning of *IscU*
3NdeI-IscU-CStrep	CCTCATATGAACCAGGCCTTTCTTGTGCTT	Cloning of *IscU*
